# 2-(2,3-Dioxoindolin-1-yl)ethyl 4-(4-nitro­phen­yl)piperazine-1-carbodithio­ate

**DOI:** 10.1107/S1600536810030618

**Published:** 2010-08-11

**Authors:** Yao Wang, Chong-Qing Wan, Sheng-Li Cao, Tingting Zheng

**Affiliations:** a Department of Chemistry, Capital Normal University, Beijing 100048, People’s Republic of China

## Abstract

In the title compound, C_21_H_20_N_4_O_4_S_2, _the piperazine ring adopts a chair conformation. The 1-ethyl­indoline-2,3-dione system links to one N atom of the piperazine ring *via* a carbodithio­ate group. The indoline-2,3-dione ring and the nitro­benzene ring subtend adihedral angle of 37.27 (7)°. In the crystal structure, weak C—H⋯O and π–π stacking inter­actions [centroid–centroid distances = 3.534 (5) and 3.797 (5) Å] may help to establish the packing.

## Related literature

For the fungicidal activity of dithio­carbamates, see: Farghaly *et al.* (1999 ▶); Xu *et al.* (2002 ▶); Ozkirimli *et al.* (2005 ▶) and for their anti­bacterial activity, see: Chourasia *et al.* (1999 ▶); Imamura *et al.* (2001 ▶). For the effective anti­tumor activity of dithio­carbamates, see: Cao *et al.* (2005 ▶); Gaspari *et al.* (2006 ▶). For a description of the Cambridge Structural Database, see: Allen (2002 ▶).
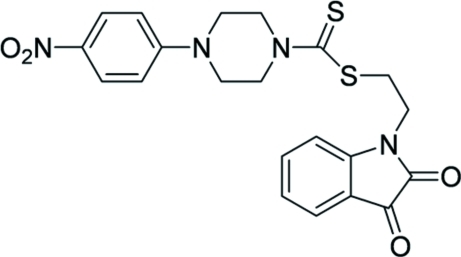


## Experimental

### 

#### Crystal data

C_21_H_20_N_4_O_4_S_2_
*M* *_r_* = 456.53
Monoclinic, 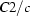

*a* = 34.4244 (8) Å
*b* = 6.8754 (2) Å
*c* = 17.9938 (4) Å
β = 103.185 (1)°
*V* = 4146.53 (18) Å^3^
*Z* = 8
Mo *K*α radiation
μ = 0.29 mm^−1^
*T* = 296 K
0.15 × 0.13 × 0.10 mm

#### Data collection

Bruker APEXII CCD area-detector diffractometer
25340 measured reflections
5843 independent reflections
4309 reflections with *I* > 2σ(*I*)
*R*_int_ = 0.027

#### Refinement

*R*[*F* ^2^ > 2σ(*F* ^2^)] = 0.039
*wR*(*F* ^2^) = 0.107
*S* = 1.03
5843 reflections
280 parameters
H-atom parameters constrained
Δρ_max_ = 0.27 e Å^−3^
Δρ_min_ = −0.18 e Å^−3^

### 

Data collection: *APEX2* (Bruker, 2007 ▶); cell refinement: *APEX2* and *SAINT* (Bruker, 2007 ▶); data reduction: *SAINT*; program(s) used to solve structure: *SHELXS97* (Sheldrick, 2008 ▶); program(s) used to refine structure: *SHELXL97* (Sheldrick, 2008 ▶); molecular graphics: *SHELXTL* (Sheldrick, 2008 ▶); software used to prepare material for publication: *SHELXTL* and *PLATON* (Spek, 2009 ▶).

## Supplementary Material

Crystal structure: contains datablocks I, global. DOI: 10.1107/S1600536810030618/zq2052sup1.cif

Structure factors: contains datablocks I. DOI: 10.1107/S1600536810030618/zq2052Isup2.hkl

Additional supplementary materials:  crystallographic information; 3D view; checkCIF report

## Figures and Tables

**Table 1:** Hydrogen-bond geometry (Å, °)

*D*—H⋯*A*	*D*—H	H⋯*A*	*D*⋯*A*	*D*—H⋯*A*
C13—H13⋯O2^i^	0.93	2.52	3.252 (2)	136
C5—H5⋯O3^ii^	0.93	2.33	3.125 (2)	143

Symmetry codes: (i) 
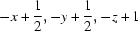
; (ii) 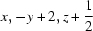.

**Fig. 1.**
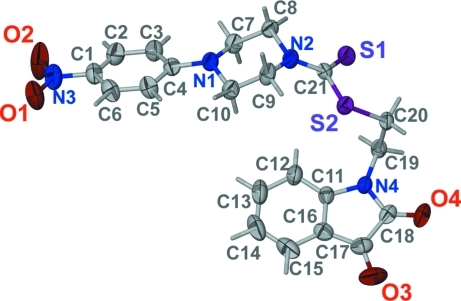
The title molecule with the atomic numbering scheme. The displacement ellipsoids are shown at the 50% probability level.

**Fig. 2.**
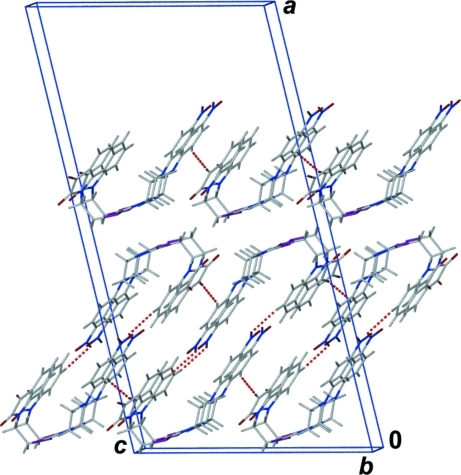
The packing structure of the title compound. The red dashed lines indicate the intermolecular C—H···O interactions, while the π–π stacking interactions are omitted for clarity.

## supplementary crystallographic information

### Comment

Dithiocarbamates represent a broad spectrum of biological activities such as fungicidal (Farghaly *et al.*, 1999; Xu *et al.*, 2002; Ozkirimli *et al.*, 2005) and antibacterial effects (Chourasia *et al.*, 1999; Imamura *et al.*, 2001). Dithiocarbamates were also proved to have *in vitro* and *in vivo* effective antitumor activities (Cao *et al.*, 2005; Gaspari *et al.*, 2006). In an effort to obtain new and more potent antibacterial and antitumor compounds, we synthesized the title compound, a kind of novel dithiocarbamate derivative. Here, we report the crystal structure of the title compound.

The X-ray crystal analysis shown that the piperazine ring adopts a chair conformation. The indole-2,3-dione ring and the nitrobenzene ring exhibit a dihedral angle of 37.27 (7)° (Fig.1). Considering the molecule with a U shape conformation, two molecules arrange in a face-to-face mode interacting through intermolecular hydrogen bonding (C13—H13···O2^i^ (nitro) = 2.52 Å, D···A 3.252 (2) Å, D—H···A 136.2°). The dimers are further stacked through π–π and C—H···O intermolecular interactions: C5—H5···O3^ii^ (dione) = 2.33 Å (D···A 3.125 (1) Å, D—H···A 143.3°) and the centroid-centroid distance between the C1—C2—C3—C4—C5—C6 (benzene) and C11^iii^—C16^iii^—C17^iii^—C18^iii^—N4^iii^ (pyrrole) rings is 3.534 (5) Å, and the distance between the C1—C2—C3—C4—C5—C6 (benzene) and C11^iii^—C12^iii^—C13^iii^—C14^iii^—C15^iii^—C16^iii^ rings is 3.797 (5) Å. The packing structure is shown along the *a* axis in Fig.2 [symmetry codes: (i) –x+0.5, -*y* + 1/2, -*z* + 1; (ii) *x*, -*y* + 2, *z* + 1/2; (iii) *x*, -*y* + 1, *z* + 1/2].

### Experimental

A suspension of 1-(4-nitrophenyl) piperazine (2.4 mmol), carbon disulfide (0.72 ml,12 mmol) and anhydrous potassium phosphate (0.51 g, 2.4 mmol) in *N*,*N*-dimethylformamide (15 ml) was stirred at room temperature for 30 min. Then, 1-(2-bromoethyl) indoline-2,3-dione (2 mmol) was added and the stirring was continued for 30 min. The reaction mixture was poured into water (100 ml) and the resulting precipitate was separated by filtration and purified by column chromatography (CC) on silica gel with dichloromethane/methanol = 95:5, *v*/*v*, as an eluent, to give the title compound (*R_f_* = 0.79, dichloromethane/methanol = 95:5, *v*/*v*; m.p. 252–254°C; yield 90.6%). The orange crystals of the title compound were obtained by slow evaporation from a solution of dichloromethane/*N*,*N*-dimethylformamide 50:50 (*v*/*v*) at room temperature.

### Refinement

All the H atoms were discernible in the difference electron density maps. Nevertheless, the hydrogen atoms were placed into idealized positions and allowed to ride on the carrier atoms, with C—H = 0.93 and 0.97 Å for aryl and methylene H atoms, respectively, and with *U_iso_*(H) = 1.2*U_eq_*(C).

### Crystal data

**Table:**

C_21_H_20_N_4_O_4_S_2_	*F*(000) = 1904
*M**_r_* = 456.53	*D*_x_ = 1.463 Mg m^−^^3^*D*_m_ = 1.463 Mg m^−^^3^*D*_m_ measured by not measured
Monoclinic, *C*2/*c*	Mo *K*α radiation, λ = 0.71073 Å
*a* = 34.4244 (8) Å	Cell parameters from 8247 reflections
*b* = 6.8754 (2) Å	θ = 2.3–29.2°
*c* = 17.9938 (4) Å	µ = 0.29 mm^−^^1^
β = 103.185 (1)°	*T* = 296 K
*V* = 4146.53 (18) Å^3^	Block, orange
*Z* = 8	0.15 × 0.13 × 0.10 mm

### Data collection

**Table:**

Bruker APEXII CCD area-detector diffractometer	4309 reflections with *I* > 2σ(*I*)
Radiation source: fine-focus sealed tube	*R*_int_ = 0.027
graphite	θ_max_ = 29.8°, θ_min_ = 2.3°
phi and ω scans	*h* = −45→48
25340 measured reflections	*k* = −9→7
5843 independent reflections	*l* = −24→22

### Refinement

**Table:**

Refinement on *F*^2^	Primary atom site location: structure-invariant direct methods
Least-squares matrix: full	Secondary atom site location: difference Fourier map
*R*[*F*^2^ > 2σ(*F*^2^)] = 0.039	Hydrogen site location: inferred from neighbouring sites
*wR*(*F*^2^) = 0.107	H-atom parameters constrained
*S* = 1.03	*w* = 1/[σ^2^(*F*_o_^2^) + (0.0528*P*)^2^ + 1.1993*P*] where *P* = (*F*_o_^2^ + 2*F*_c_^2^)/3
5843 reflections	(Δ/σ)_max_ < 0.001
280 parameters	Δρ_max_ = 0.27 e Å^−^^3^
0 restraints	Δρ_min_ = −0.18 e Å^−^^3^

### Special details

**Table:**

Geometry. All e.s.d.'s (except the e.s.d. in the dihedral angle between two l.s. planes) are estimated using the full covariance matrix. The cell e.s.d.'s are taken into account individually in the estimation of e.s.d.'s in distances, angles and torsion angles; correlations between e.s.d.'s in cell parameters are only used when they are defined by crystal symmetry. An approximate (isotropic) treatment of cell e.s.d.'s is used for estimating e.s.d.'s involving l.s. planes.
Refinement. Refinement of *F*^2^ against ALL reflections. The weighted *R*-factor *wR* and goodness of fit *S* are based on *F*^2^, conventional *R*-factors *R* are based on *F*, with *F* set to zero for negative *F*^2^. The threshold expression of *F*^2^ > σ(*F*^2^) is used only for calculating *R*-factors(gt) *etc*. and is not relevant to the choice of reflections for refinement. *R*-factors based on *F*^2^ are statistically about twice as large as those based on *F*, and *R*- factors based on ALL data will be even larger.

### Fractional atomic coordinates and isotropic or equivalent isotropic displacement parameters (Å^2^)

**Table:**

	*x*	*y*	*z*	*U*_iso_*/*U*_eq_	
C1	0.22169 (4)	0.0960 (3)	0.55107 (9)	0.0525 (4)	
C2	0.20345 (5)	−0.0339 (3)	0.49616 (10)	0.0586 (4)	
H2	0.2133	−0.1597	0.4960	0.070*	
C3	0.17055 (5)	0.0221 (2)	0.44143 (9)	0.0522 (4)	
H3	0.1585	−0.0664	0.4042	0.063*	
C4	0.15485 (4)	0.2101 (2)	0.44084 (8)	0.0418 (3)	
C5	0.17505 (5)	0.3391 (3)	0.49705 (9)	0.0529 (4)	
H5	0.1660	0.4664	0.4974	0.063*	
C6	0.20780 (5)	0.2823 (3)	0.55145 (10)	0.0561 (4)	
H6	0.2205	0.3700	0.5884	0.067*	
C7	0.10263 (5)	0.1336 (2)	0.32661 (9)	0.0469 (3)	
H7A	0.1046	0.0009	0.3453	0.056*	
H7B	0.1175	0.1426	0.2870	0.056*	
C8	0.05960 (5)	0.1827 (2)	0.29344 (9)	0.0476 (3)	
H8A	0.0493	0.0991	0.2500	0.057*	
H8B	0.0441	0.1595	0.3313	0.057*	
C9	0.07011 (5)	0.5142 (2)	0.33419 (8)	0.0499 (4)	
H9A	0.0544	0.4955	0.3721	0.060*	
H9B	0.0673	0.6487	0.3175	0.060*	
C10	0.11353 (5)	0.4710 (2)	0.36929 (9)	0.0509 (4)	
H10A	0.1295	0.5070	0.3335	0.061*	
H10B	0.1222	0.5493	0.4149	0.061*	
C11	0.12259 (4)	0.8667 (2)	0.12798 (7)	0.0400 (3)	
C12	0.13746 (5)	0.7170 (3)	0.17715 (9)	0.0526 (4)	
H12	0.1250	0.5962	0.1733	0.063*	
C13	0.17187 (5)	0.7545 (4)	0.23274 (10)	0.0698 (6)	
H13	0.1826	0.6558	0.2666	0.084*	
C14	0.19059 (5)	0.9315 (4)	0.23961 (11)	0.0757 (6)	
H14	0.2135	0.9509	0.2779	0.091*	
C15	0.17588 (5)	1.0806 (3)	0.19041 (11)	0.0672 (5)	
H15	0.1885	1.2012	0.1948	0.081*	
C16	0.14155 (4)	1.0463 (2)	0.13361 (9)	0.0483 (4)	
C17	0.12020 (5)	1.1645 (2)	0.07061 (10)	0.0509 (4)	
C18	0.08641 (4)	1.0341 (2)	0.02587 (8)	0.0436 (3)	
C19	0.06096 (4)	0.7055 (2)	0.04397 (8)	0.0415 (3)	
H19A	0.0745	0.5833	0.0592	0.050*	
H19B	0.0517	0.7040	−0.0111	0.050*	
C20	0.02520 (4)	0.7204 (2)	0.07963 (8)	0.0411 (3)	
H20A	0.0061	0.6212	0.0573	0.049*	
H20B	0.0126	0.8459	0.0665	0.049*	
C22	0.04231 (4)	0.4390 (2)	0.19615 (7)	0.0368 (3)	
N1	0.12011 (4)	0.26548 (18)	0.38917 (7)	0.0420 (3)	
N2	0.05537 (4)	0.38618 (19)	0.26924 (7)	0.0446 (3)	
N3	0.25633 (5)	0.0353 (3)	0.60869 (9)	0.0688 (4)	
N4	0.08920 (3)	0.86365 (18)	0.06542 (6)	0.0386 (3)	
O1	0.27296 (4)	0.1532 (3)	0.65616 (9)	0.0878 (5)	
O2	0.26757 (6)	−0.1329 (3)	0.60752 (10)	0.1117 (7)	
O3	0.12620 (4)	1.32892 (19)	0.05188 (9)	0.0767 (4)	
O4	0.06274 (4)	1.07378 (18)	−0.03291 (6)	0.0592 (3)	
S1	0.031276 (12)	0.28227 (6)	0.12387 (2)	0.04687 (11)	
S2	0.036236 (11)	0.69395 (5)	0.18228 (2)	0.04120 (10)	

### Atomic displacement parameters (Å^2^)

**Table:**

	*U*^11^	*U*^22^	*U*^33^	*U*^12^	*U*^13^	*U*^23^
C1	0.0424 (8)	0.0715 (12)	0.0423 (8)	0.0063 (8)	0.0071 (6)	0.0042 (8)
C2	0.0598 (10)	0.0559 (11)	0.0567 (10)	0.0124 (8)	0.0061 (8)	0.0048 (8)
C3	0.0558 (9)	0.0501 (9)	0.0463 (8)	0.0047 (7)	0.0025 (7)	−0.0024 (7)
C4	0.0403 (7)	0.0501 (9)	0.0361 (7)	0.0013 (6)	0.0108 (6)	0.0001 (6)
C5	0.0470 (8)	0.0566 (10)	0.0516 (9)	0.0072 (7)	0.0043 (7)	−0.0127 (8)
C6	0.0455 (8)	0.0718 (12)	0.0479 (9)	0.0012 (8)	0.0047 (7)	−0.0132 (8)
C7	0.0557 (8)	0.0401 (8)	0.0420 (8)	0.0016 (7)	0.0051 (6)	−0.0050 (6)
C8	0.0551 (8)	0.0400 (8)	0.0429 (8)	−0.0050 (7)	0.0011 (6)	0.0027 (6)
C9	0.0687 (10)	0.0426 (9)	0.0328 (7)	0.0089 (7)	−0.0002 (6)	−0.0043 (6)
C10	0.0633 (9)	0.0414 (8)	0.0417 (8)	−0.0043 (7)	−0.0011 (7)	−0.0021 (7)
C11	0.0381 (7)	0.0504 (8)	0.0315 (7)	0.0028 (6)	0.0078 (5)	0.0015 (6)
C12	0.0474 (8)	0.0650 (11)	0.0425 (8)	0.0055 (7)	0.0040 (6)	0.0119 (7)
C13	0.0485 (9)	0.1117 (17)	0.0451 (9)	0.0125 (10)	0.0025 (7)	0.0187 (10)
C14	0.0451 (9)	0.130 (2)	0.0468 (10)	−0.0066 (11)	−0.0011 (7)	−0.0083 (11)
C15	0.0524 (10)	0.0881 (15)	0.0624 (11)	−0.0210 (10)	0.0160 (8)	−0.0200 (10)
C16	0.0455 (8)	0.0548 (10)	0.0470 (8)	−0.0061 (7)	0.0158 (6)	−0.0067 (7)
C17	0.0543 (9)	0.0430 (9)	0.0628 (10)	0.0008 (7)	0.0289 (8)	0.0016 (7)
C18	0.0490 (8)	0.0447 (8)	0.0405 (7)	0.0114 (6)	0.0177 (6)	0.0067 (6)
C19	0.0469 (7)	0.0418 (8)	0.0329 (7)	0.0010 (6)	0.0029 (5)	−0.0030 (6)
C20	0.0401 (7)	0.0442 (8)	0.0348 (7)	0.0019 (6)	−0.0005 (5)	0.0030 (6)
C22	0.0335 (6)	0.0390 (7)	0.0363 (7)	−0.0002 (5)	0.0048 (5)	−0.0025 (5)
N1	0.0470 (6)	0.0385 (7)	0.0374 (6)	0.0021 (5)	0.0032 (5)	−0.0027 (5)
N2	0.0545 (7)	0.0387 (7)	0.0357 (6)	0.0014 (5)	0.0000 (5)	−0.0014 (5)
N3	0.0573 (9)	0.0930 (14)	0.0513 (9)	0.0138 (9)	0.0020 (7)	0.0054 (9)
N4	0.0404 (6)	0.0400 (6)	0.0333 (6)	0.0021 (5)	0.0042 (5)	0.0037 (5)
O1	0.0680 (8)	0.1118 (13)	0.0681 (9)	0.0036 (9)	−0.0170 (7)	−0.0075 (9)
O2	0.1188 (14)	0.1077 (14)	0.0826 (11)	0.0530 (12)	−0.0310 (10)	−0.0040 (10)
O3	0.0829 (9)	0.0482 (7)	0.1107 (11)	−0.0045 (7)	0.0461 (9)	0.0118 (7)
O4	0.0647 (7)	0.0675 (8)	0.0443 (6)	0.0220 (6)	0.0101 (5)	0.0179 (5)
S1	0.0547 (2)	0.0437 (2)	0.0383 (2)	−0.00326 (16)	0.00241 (15)	−0.00880 (15)
S2	0.0496 (2)	0.0381 (2)	0.03489 (18)	0.00262 (14)	0.00753 (14)	−0.00231 (14)

### Geometric parameters (Å, °)

**Table:**

C1—C6	1.368 (3)	C11—C16	1.390 (2)
C1—C2	1.373 (2)	C11—N4	1.4136 (18)
C1—N3	1.452 (2)	C12—C13	1.389 (2)
C2—C3	1.375 (2)	C12—H12	0.9300
C2—H2	0.9300	C13—C14	1.369 (3)
C3—C4	1.400 (2)	C13—H13	0.9300
C3—H3	0.9300	C14—C15	1.374 (3)
C4—N1	1.3899 (19)	C14—H14	0.9300
C4—C5	1.404 (2)	C15—C16	1.395 (2)
C5—C6	1.371 (2)	C15—H15	0.9300
C5—H5	0.9300	C16—C17	1.451 (2)
C6—H6	0.9300	C17—O3	1.211 (2)
C7—N1	1.4635 (19)	C17—C18	1.542 (2)
C7—C8	1.504 (2)	C18—O4	1.2097 (17)
C7—H7A	0.9700	C18—N4	1.3633 (19)
C7—H7B	0.9700	C19—N4	1.4511 (19)
C8—N2	1.462 (2)	C19—C20	1.516 (2)
C8—H8A	0.9700	C19—H19A	0.9700
C8—H8B	0.9700	C19—H19B	0.9700
C9—N2	1.4585 (18)	C20—S2	1.8080 (14)
C9—C10	1.513 (2)	C20—H20A	0.9700
C9—H9A	0.9700	C20—H20B	0.9700
C9—H9B	0.9700	C22—N2	1.3394 (17)
C10—N1	1.4624 (19)	C22—S1	1.6650 (14)
C10—H10A	0.9700	C22—S2	1.7760 (14)
C10—H10B	0.9700	N3—O2	1.221 (2)
C11—C12	1.377 (2)	N3—O1	1.221 (2)
			
C6—C1—C2	120.64 (15)	C13—C12—H12	121.5
C6—C1—N3	119.78 (16)	C14—C13—C12	122.45 (19)
C2—C1—N3	119.58 (17)	C14—C13—H13	118.8
C1—C2—C3	120.01 (17)	C12—C13—H13	118.8
C1—C2—H2	120.0	C13—C14—C15	120.56 (17)
C3—C2—H2	120.0	C13—C14—H14	119.7
C2—C3—C4	121.14 (16)	C15—C14—H14	119.7
C2—C3—H3	119.4	C14—C15—C16	118.17 (19)
C4—C3—H3	119.4	C14—C15—H15	120.9
N1—C4—C3	121.97 (14)	C16—C15—H15	120.9
N1—C4—C5	121.19 (14)	C11—C16—C15	120.60 (17)
C3—C4—C5	116.79 (14)	C11—C16—C17	107.15 (14)
C6—C5—C4	121.74 (16)	C15—C16—C17	132.16 (17)
C6—C5—H5	119.1	O3—C17—C16	130.71 (17)
C4—C5—H5	119.1	O3—C17—C18	123.61 (17)
C1—C6—C5	119.65 (16)	C16—C17—C18	105.65 (13)
C1—C6—H6	120.2	O4—C18—N4	127.11 (15)
C5—C6—H6	120.2	O4—C18—C17	127.08 (15)
N1—C7—C8	111.19 (13)	N4—C18—C17	105.80 (12)
N1—C7—H7A	109.4	N4—C19—C20	113.34 (12)
C8—C7—H7A	109.4	N4—C19—H19A	108.9
N1—C7—H7B	109.4	C20—C19—H19A	108.9
C8—C7—H7B	109.4	N4—C19—H19B	108.9
H7A—C7—H7B	108.0	C20—C19—H19B	108.9
N2—C8—C7	110.79 (13)	H19A—C19—H19B	107.7
N2—C8—H8A	109.5	C19—C20—S2	115.08 (10)
C7—C8—H8A	109.5	C19—C20—H20A	108.5
N2—C8—H8B	109.5	S2—C20—H20A	108.5
C7—C8—H8B	109.5	C19—C20—H20B	108.5
H8A—C8—H8B	108.1	S2—C20—H20B	108.5
N2—C9—C10	110.25 (13)	H20A—C20—H20B	107.5
N2—C9—H9A	109.6	N2—C22—S1	123.85 (11)
C10—C9—H9A	109.6	N2—C22—S2	114.09 (10)
N2—C9—H9B	109.6	S1—C22—S2	122.04 (8)
C10—C9—H9B	109.6	C4—N1—C10	119.33 (12)
H9A—C9—H9B	108.1	C4—N1—C7	119.01 (12)
N1—C10—C9	112.02 (13)	C10—N1—C7	113.37 (12)
N1—C10—H10A	109.2	C22—N2—C9	126.91 (13)
C9—C10—H10A	109.2	C22—N2—C8	122.69 (12)
N1—C10—H10B	109.2	C9—N2—C8	110.23 (11)
C9—C10—H10B	109.2	O2—N3—O1	122.74 (18)
H10A—C10—H10B	107.9	O2—N3—C1	118.16 (18)
C12—C11—C16	121.17 (14)	O1—N3—C1	119.10 (18)
C12—C11—N4	128.02 (15)	C18—N4—C11	110.53 (12)
C16—C11—N4	110.75 (13)	C18—N4—C19	122.84 (12)
C11—C12—C13	117.04 (18)	C11—N4—C19	126.64 (12)
C11—C12—H12	121.5	C22—S2—C20	103.51 (7)
			
C6—C1—C2—C3	−0.9 (3)	C5—C4—N1—C10	27.1 (2)
N3—C1—C2—C3	179.84 (16)	C3—C4—N1—C7	−9.1 (2)
C1—C2—C3—C4	−0.6 (3)	C5—C4—N1—C7	173.37 (14)
C2—C3—C4—N1	−175.64 (15)	C9—C10—N1—C4	−161.47 (12)
C2—C3—C4—C5	2.0 (2)	C9—C10—N1—C7	50.45 (17)
N1—C4—C5—C6	175.60 (15)	C8—C7—N1—C4	161.00 (13)
C3—C4—C5—C6	−2.0 (2)	C8—C7—N1—C10	−50.81 (18)
C2—C1—C6—C5	0.8 (3)	S1—C22—N2—C9	172.05 (12)
N3—C1—C6—C5	−179.89 (15)	S2—C22—N2—C9	−9.64 (19)
C4—C5—C6—C1	0.7 (3)	S1—C22—N2—C8	−2.8 (2)
N1—C7—C8—N2	55.31 (17)	S2—C22—N2—C8	175.50 (11)
N2—C9—C10—N1	−54.06 (17)	C10—C9—N2—C22	−116.39 (16)
C16—C11—C12—C13	0.6 (2)	C10—C9—N2—C8	58.99 (17)
N4—C11—C12—C13	177.47 (15)	C7—C8—N2—C22	115.48 (15)
C11—C12—C13—C14	0.2 (3)	C7—C8—N2—C9	−60.14 (16)
C12—C13—C14—C15	−0.5 (3)	C6—C1—N3—O2	178.82 (19)
C13—C14—C15—C16	0.0 (3)	C2—C1—N3—O2	−1.9 (3)
C12—C11—C16—C15	−1.2 (2)	C6—C1—N3—O1	−1.1 (3)
N4—C11—C16—C15	−178.54 (13)	C2—C1—N3—O1	178.16 (17)
C12—C11—C16—C17	175.79 (13)	O4—C18—N4—C11	175.30 (14)
N4—C11—C16—C17	−1.57 (16)	C17—C18—N4—C11	−3.55 (14)
C14—C15—C16—C11	0.9 (2)	O4—C18—N4—C19	−4.8 (2)
C14—C15—C16—C17	−175.21 (16)	C17—C18—N4—C19	176.33 (12)
C11—C16—C17—O3	−178.79 (16)	C12—C11—N4—C18	−173.73 (14)
C15—C16—C17—O3	−2.3 (3)	C16—C11—N4—C18	3.40 (16)
C11—C16—C17—C18	−0.57 (15)	C12—C11—N4—C19	6.4 (2)
C15—C16—C17—C18	175.91 (16)	C16—C11—N4—C19	−176.48 (13)
O3—C17—C18—O4	2.1 (2)	C20—C19—N4—C18	−88.55 (15)
C16—C17—C18—O4	−176.31 (14)	C20—C19—N4—C11	91.31 (16)
O3—C17—C18—N4	−179.08 (15)	N2—C22—S2—C20	172.63 (10)
C16—C17—C18—N4	2.54 (14)	S1—C22—S2—C20	−9.02 (10)
N4—C19—C20—S2	−66.16 (15)	C19—C20—S2—C22	−77.72 (12)
C3—C4—N1—C10	−155.40 (14)		

### Hydrogen-bond geometry (Å, °)

**Table:**

*D*—H···*A*	*D*—H	H···*A*	*D*···*A*	*D*—H···*A*
C13—H13···O2^i^	0.93	2.52	3.252 (2)	136.
C5—H5···O3^ii^	0.93	2.33	3.125 (2)	143.

Symmetry codes: (i) −*x*+1/2, −*y*+1/2, −*z*+1; (ii) *x*, −*y*+2, *z*+1/2.

### Table 2 π–π interactions (Å)'

**Table:**

Cg	Cg	Cg···Cg (Å)	sym. code
Cg1	Cg2	3.534 (5)	x, -y+1, z+0.5
Cg1	Cg3	3.797 (5)	x, -y+1, z+0.5

^*^ Cg1, Cg2 and Cg3 are the centroids of the C1-C2-C3-C4-C5-C6 (benzene), C11-C16-C17-C18-N4 (pyrrole) and C11-C12-C13-C14-C15-C16 rings, respectively.

## References

[bb1] Allen, F. H. (2002). *Acta Cryst.* B**58**, 380–388. 10.1107/s0108768102003890 12037359

[bb2] Bruker (2007). *APEX2*, *SADABS* and *SAINT* Bruker AXS Inc., Madison, Wisconsin, USA.

[bb3] Cao, S. L., Feng, Y. P., Jiang, Y. Y., Liu, S. Y., Ding, G. Y. & Li, R. T. (2005). *Bioorg. Med. Chem. Lett.* **15**, 1915–1917. 10.1016/j.bmcl.2005.01.083 15780632

[bb4] Chourasia, M. R. & Tyagi, D. (1999). *Indian J. Phy. Nat. Sci.* **15**, 15–21.

[bb5] Farghaly, A. O. & Moharram, A. M. (1999). *Boll. Chim. Farmaceut.* **138**, 280–289. 10464978

[bb6] Gaspari, P., Banerjee, T., Malachowski, W. P., Muller, A. J., Prendergast, G. C., DuHadaway, J., Bennett, S. & Donovan, A. M. (2006). *J. Med. Chem.* **49**, 684–692. 10.1021/jm0508888 PMC2527235 16420054

[bb7] Imamura, H., Ohtake, N., Jona, H., Shimizu, A., Moriya, M., Sato, H., Sugimoto, Y., Ikeura, C., Kiyonaga, H., Nakano, M., Nagano, R., Abe, S., Yamada, K., Hashizume, T. & Morishima, H. (2001). *Bioorg. Med. Chem.* **9**, 1571–1578. 10.1016/s0968-0896(01)00044-x 11408176

[bb8] Ozkirimli, S., Apak, T. I., Kiraz, M. & Yegenoglu, Y. (2005). *Arch. Pharm. Res.* **28**, 1213–1218. 10.1007/BF02978200 16350843

[bb9] Sheldrick, G. M. (2008). *Acta Cryst.* A**64**, 112–122. 10.1107/S0108767307043930 18156677

[bb10] Spek, A. L. (2009). *Acta Cryst.* D**65**, 148–155. 10.1107/S090744490804362X PMC2631630 19171970

[bb11] Xu, L. Z., Jiao, K., Zhang, S. S. & Kuang, S. P. (2002). *Bull. Korean Chem. Soc.* **23**, 1699–1701.

